# Machine learning combined with body composition predicts surgical difficulty in mid-low rectal cancer surgery

**DOI:** 10.1080/07853890.2025.2582235

**Published:** 2025-11-17

**Authors:** Xiangyong Li, Xiaoyang Zhang, Chenxi Zhou, Mengchao Sheng, Tengfei He, Xiaodong Yang, Yong Wu, Chungen Xing

**Affiliations:** Department of Gastrointestinal Surgery, The Second Affiliated Hospital of Soochow University, Suzhou City, Jiangsu Province, China

**Keywords:** LaTME, machine learning, predictive model, rectal cancer, surgical difficulty

## Abstract

**Background:**

This study sought to identify critical body composition characteristics associated with surgical difficulty in Laparoscopic Total Mesorectal Excision (LaTME) and to develop and validate an interpretable machine learning model using body composition data.

**Methods:**

Patients with pathologically confirmed mid to low rectal cancer treated between January 2017 and December 2022 were enrolled. LASSO regression identified clinical features most predictive of surgical difficulty. Seven machine learning algorithms were developed and validated. Model performance was comprehensively assessed using area under the receiver operating characteristic curve (AUC), accuracy, sensitivity, Brier score, calibration curves, and decision curve analysis. SHapley Additive exPlanations (SHAP) values elucidated key feature contributions, and the optimal model was implemented as a dynamic nomogram.

**Results:**

A cohort of 387 rectal cancer patients undergoing LaTME was enrolled. LASSO regression identified the following predictors for model development: visceral fat area (VFA), visceral fat ratio (VFR), tumor anal verge distance, subcutaneous fat area (SFA), receipt of neoadjuvant therapy, neutrophil-to-lymphocyte ratio (NLR), and history of abdominal surgery. In the validation cohort, the logistic regression (LR) model demonstrated optimal performance . SHAP analysis revealed that increased VFA, elevated VFR, greater SFA, shorter tumor anal verge distance, administration of neoadjuvant therapy, higher NLR, and prior abdominal surgery were associated with increased surgical difficulty during LaTME. Kaplan–Meier analysis demonstrated significantly reduced 1-year, 3-year, and 5-year overall survival (OS) rates in the difficult surgery cohort compared to the non-difficult cohort (*p* < 0.05).

**Conclusion:**

Seven predictive models for LaTME surgical difficulty were constructed and validated. The LR model exhibited the best predictive performance. Survival analysis indicated poorer prognosis in patients experiencing difficult surgery.

## Background

Global Cancer Statistics 2022 rank colorectal cancer (CRC) as the third most prevalent malignancy worldwide and the second leading cause of cancer-related mortality. Rectal cancer (RC), constituting approximately one-third of CRC cases, presents greater clinical challenges than colon cancer due to its distinct anatomical characteristics and therapeutic complexity [1,2]. Although treatment modalities include radiotherapy, chemotherapy, and targeted therapy, surgical resection remains pivotal for early-to-mid stage and selected advanced mid-low RC [3–5].

Among surgical techniques, total mesorectal excision (TME) described by Heald significantly improved oncological outcomes [6]. LaTME has emerged as the standard surgical approach for mid-low RC, offering advantages of reduced invasiveness and accelerated recovery. Crucially, LaTME demonstrates comparable pathological safety and OS to open TME, with superior short-term outcomes [3]. However, LaTME’s inherent technical complexity increases surgical skill demands and intraoperative uncertainties [7,8], frequently resulting in unplanned conversions to open surgery and elevated postoperative complication rates, significantly impacting recovery, quality of life, and long-term prognosis [9,10].

Surgical difficulty in LaTME arises from multifactorial interactions involving surgeon experience, surgical approach, tumor location, and pathological stage [11]. Anatomical constraints including a prominent sacral promontory, acute sacral curvature, and narrow bony pelvis correlate with increased technical demands [12–14], though conflicting evidence exists regarding specific pelvic parameters [15]. These inconsistencies highlight the need for standardized surgical difficulty assessment systems.

While obesity’s impact on surgical difficulty is established, reliance on Body Mass Index (BMI) has limitations [9,10]. Increased intra-abdominal fat volume—impairing surgical field exposure and instrument maneuverability—represents a key determinant [16]. Computed Tomography (CT)-based quantification enables precise measurement of VAT and SAT cross-sectional areas, with fat quality assessment *via* mean radiodensity, demonstrating high reproducibility [17,18].

Machine Learning (ML), a core Artificial Intelligence (AI) technology, is increasingly applied to medical data analysis [19–21]. ML excels at identifying complex variable interactions within large datasets to construct highly accurate predictive models.

## Materials and methods

### Patients and ethical approval

This study collected clinicopathological data from patients who underwent LaTME at The Second Affiliated Hospital of Soochow University between January 2017 and December 2022.

Inclusion criteria:

1. Pathologically confirmed RC diagnosis before surgery;
2. Complete preoperative CT and clinical data within two weeks prior to surgery;
3. Planned LaTME procedure.

**Figure 1.**
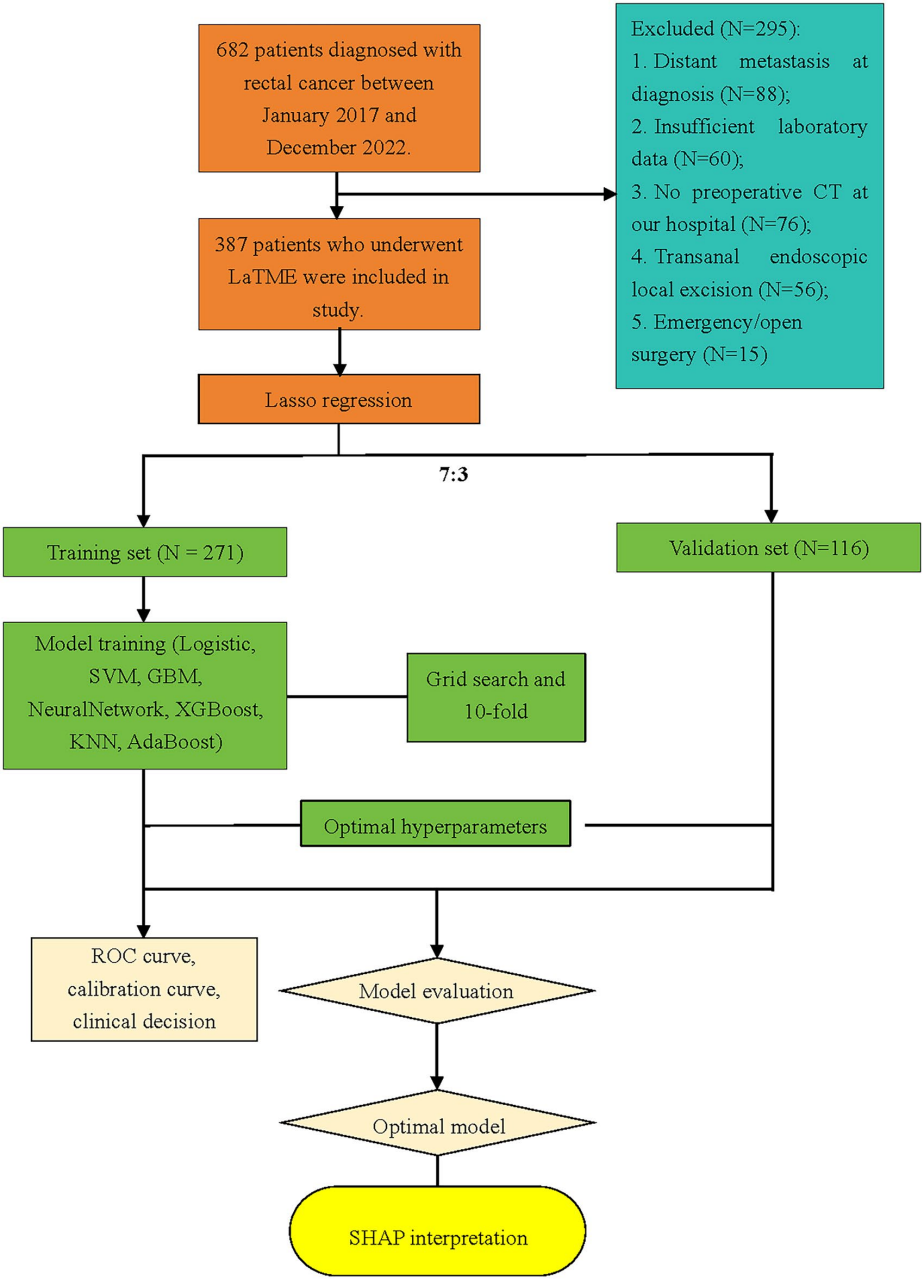
Study enrollment flowchart.

Exclusion criteria:

1. Emergency or open surgery;
2. Incomplete or excessively missing CT/clinical data;
3. Local excision via transanal endoscopy;
4. Clinical stage IV disease or tumors deemed inoperable due to excessive size (Figure 1).

Postoperative short-term complications were classified using the Clavien–Dindo classification [22].

### Data collection

The following characteristics were retrieved from the Electronic Medical Record and Surgery Anesthesia Systems at The Second Affiliated Hospital of Soochow University:

1. Baseline characteristics: Sex, age, height, weight, American Society of Anesthesiologists (ASA) score, comorbidities (hypertension, diabetes), and preoperative neoadjuvant therapy status.
2. Laboratory parameters: Albumin (Alb), Hemoglobin (Hb), Triglycerides (TG), Total cholesterol (TC), C-reactive protein (CRP), White blood cell count (×10^9^/L), Neutrophil count (×10^9^/L), Lymphocyte count (×10^9^/L), Carcinoembryonic antigen (CEA).
3. Perioperative data: Operative duration (min), Intraoperative blood loss (ml), Pathological findings, Postoperative hospital stay (days).
4. Calculated biomarkers: NLR = Neutrophil count/lymphocyte count; IBI = CRP × Neutrophil count/Lymphocyte count; NAR = Neutrophil count (×10^9^/L)/albumin (g/L); LCR = Lymphocyte count (×10^9^/L)/CRP (mg/L).

### Body composition quantification

This study utilized Slice-O-Matic software (v5.0; TomoVision) to analyze two consecutive CT axial slices at the L3 vertebral level (Preoperative CT was performed on a GE Revolution CT 128-slice. 5-millimeter slice thickness. Correlation with the endoscopic findings was made in all cases. DICOM format). Parameters for VAT, SAT, and SM were quantified and averaged [23] (Supplementary Figure S1). Tissue segmentation was performed using Hounsfield unit (HU) thresholds: VAT: (−150 to −50 HU); SAT: (−190 to −30 HU); SM: (−29 to +150 HU).

### Criteria for defining surgical difficulty

Building upon the evaluation framework introduced by Escal et al. [12]. This study developed a refined scoring system through methodological enhancements. This system integrates five parameters: surgery time, conversion to open surgery, postoperative hospital stay, blood loss, and Postoperative complications (Clavien–Dindo grade II/III). The scoring scale ranges from 0 to 10 points, with a threshold of 3 points defining surgical difficulty. Total scores ≤3 points designated the non-difficult surgery group, while scores >3 points defined the difficult surgery group (Table 1). Significant differences (*p* < 0.05) were observed between the two groups across all five evaluation metrics, as detailed in Table 2.

**Table 1.** Surgical difficulty scoring system.

Evaluation metrics	Score
Surgery time > 240 min	3
Conversion to open surgery	3
Postoperative hospital stay ≥ 10 days	2
Blood loss ≥ 200 ml	1
Postoperative complications(Clavien–Dindo II/III)	1

**Table 2.** Clinical characteristics in the surgically difficult and non-surgically difficult.

Characteristics	Total(*n* = 387)	Surgically difficult(*n* = 87)	Non-surgically difficult(*n* = 300)	*P*
**Baseline characteristics**				
Age (years), mean [SD]	65.44 ± 10.83	67.15 ± 11.40	64.95 ± 10.63	0.095
BMI (kg/m^2^), mean [SD]	23.73 ± 3.71	24.42 ± 4.17	23.53 ± 3.55	0.047
Sex, *n* (%)				0.199
Female	161(41.60)	31(35.63)	130(43.33)	
Male	226(58.40)	56(64.37)	170(56.67)	
Hypertension, *n* (%)				0.334
No	200(51.68)	41(47.13)	159(53.00)	
Yes	187(48.32)	46(52.87)	141(47.00)	
Diabetes, *n* (%)				0.445
No	326(84.24)	71(81.61)	255(85.00)	
Yes	61(15.76)	16(18.39)	45(15.00)	
Prior of abdominal surgery, *n* (%)				0.021
No	294(75.97)	58(66.67)	236(78.67)	
Yes	93(24.03)	29(33.33)	64(21.33)	
Neoadjuvant therapy, *n* (%)				0.008
No	349(90.18)	72(82.76)	277(92.33)	
Yes	38(9.82)	15(17.24)	23(7.67)	
ASA, *n* (%)				0.683
1	138(35.66)	110(36.67)	28(32.18)	
2	154(39.79)	119(39.67)	35(40.23)	
3	78(20.16)	57(19.00)	21(24.14)	
4	17(4.39)	14(4.67)	3(3.45)	
**Laboratory parameters**				
Hb(g/L), mean [SD]	128.90 ± 19.59	130.68 ± 21.85	128.38 ± 18.89	0.337
Alb(g/L), mean [SD]	41.41 ± 4.92	40.25 ± 6.10	41.75 ± 4.47	0.035
NLR, mean [SD]	3.05 ± 3.91	3.89 ± 5.42	2.81 ± 3.32	0.023
IBI, median [IQR]	12.57(9.21,18.84)	12.77(10.02,20.98)	12.48(9.07,18.40)	0.121
NAR, median [IQR]	0.08(0.06,0.11)	0.08(0.06,0.11)	0.08(0.06,0.11)	0.84
LCR, median [IQR]	0.26(0.19,0.36)	0.24(0.17,0.36)	0.27(0.19,0.36)	0.213
Tc (mmol/L), median [IQR]	4.59(4.12,5.22)	4.60(4.16,5.22)	4.59(4.12,5.24)	0.911
Tg (mmol/L), median [IQR]	1.18(0.91,1.54)	1.22(0.99,1.68)	1.17(0.90,1.52)	0.313
CRP, median [IQR]	5.40(5.10,5.70)	5.50(5.15,5.90)	5.40(5.10,5.70)	0.274
CEA (ng/ml), median [IQR]	3.34(2.21,6.15)	3.73(2.23,6.08)	3.24(2.21,6.19)	0.603
**Quantitatively measured CT parameters**				
SMR (HU), mean [SD]	32.78 ± 7.03	31.67 ± 8.37	33.11 ± 6.57	0.142
SFR (HU), mean [SD]	−97.40 ± 9.73	−98.58 ± 7.98	−97.06 ± 10.17	0.202
VFR (HU), mean [SD]	−92.99 ± 8.07	−93.22 ± 8.04	−92.92 ± 8.09	0.761
SMA (cm^2^), mean [SD]	118.81 ± 26.41	123.73 ± 29.19	117.39 ± 25.43	0.049
SFA (cm^2^), mean [SD]	124.31 ± 57.91	149.36 ± 67.64	117.04 ± 52.70	<0.001
VFA (cm^2^), mean [SD]	107.89 ± 59.37	146.77 ± 70.14	96.61 ± 50.67	<0.001
Tumor distance from the anal verge	8.78 ± 2.93	6.89 ± 2.78	9.33 ± 2.75	<0.001
(cm), mean [SD]				
**Pathological Characteristics**				
Differentiation grade, (*n* (%))				0.705
Well-differentiated	15(3.88)	4(4.60)	11(3.67)	
Moderately differentiated	313(80.88)	72(82.76)	241(80.33)	
Poorly differentiated	59(15.25)	11(12.64)	48(16.00)	
Perineural invasion (*n* (%))				0.744
No	316(81.65)	70(80.46)	246(82.00)	
Yes	71(18.35)	17(19.54)	54(18.00)	
Lymphovascular invasion, (*n* (%))				0.101
No	325(83.98)	78(89.66)	247(82.33)	
Yes	62(16.02)	9(10.34)	53(17.67)	
T stage, (*n* (%))				0.979
1	150(38.76)	34(39.08)	116(38.67)	
2	90(23.26)	19(21.84)	71(23.67)	
3	119(30.75)	28(32.18)	91(30.33)	
4	28(7.24)	6(6.90)	22(7.33)	
N stage, (*n* (%))				0.912
0	240(62.02)	53(60.92)	187(62.33)	
1	86(22.22)	19(21.84)	67(22.33)	
2	61(15.76)	15(17.24)	46(15.33)	
TNM stage, (*n* (%))				0.939
I	164(42.38)	37(42.53)	127(42.33)	
II	76(19.64)	16(18.39)	60(20.00)	
III	147(37.98)	34(39.08)	113(37.67)	
LNR, median [IQR]	0.00(0.00,0.13)	0.00(0.00,0.14)	0.00(0.00,0.12)	0.661
Tumor size(cm), mean [SD]	37.51 ± 15.09	38.15 ± 15.90	37.33 ± 14.87	0.656
**Surgical difficulty**				
Blood loss (ml),(*n* (%))				<0.001
≤200	347(89.66)	68(78.16)	279(93.00)	
>200	40(10.34)	19(21.84)	21(7.00)	
Conversion to open surgery, (*n* (%))				<0.001
No	365(94.32)	68(78.16)	297(99.00)	
Yes	22(5.68)	19(21.84)	3(1.00)	
Postoperative hospital stay(day), (*n* (%))				<0.001
≤10	235(60.72)	6(6.90)	229(76.33)	
>10	152(39.28)	81(93.10)	71(23.67)	
Postoperative complications, (*n* (%))				<0.001
No	334(86.30)	55(63.22)	279(93.00)	
Yes	53(13.70)	32(36.78)	21(7.00)	
Operative time(min), (*n* (%))				<0.001
≤240	236(60.98)	3(3.45)	233(77.67)	
>240	151(39.02)	84(96.55)	67(22.33)	

VFA, visceral fat area; VFR visceral fat radiodensity; SFA, subcutaneous fat area; NLR, neutrophil-to-lymphocyte ratio; CT, computed tomography; IBI, inflammatory burden index; NAR, neutrophil-to-albumin ratio; LCR, lymphocyte-to-C-reactive protein ratio; ASA, American Society of Anesthesiologists; ALB, albumin; Tg, triglycerides; Tc, total cholesterol; CRP, C-reactive protein; CEA carcinoembryonic antigen; SM, skeletal muscle.

### Follow-up

This study employed a dynamic follow-up protocol integrating clinic visits and telephone assessments. Initial follow-up occurred at 1 month postoperatively, with subsequent evaluations at 2- to 3-month intervals during the first two postoperative years. Beginning in year 3, follow-ups transitioned to 6-month intervals through year 5. Beyond five years, surveillance continued annually. Follow-up concluded upon reaching the June 2024 study endpoint or patient death. The primary endpoint was OS, calculated from surgery date to last follow-up or death from any cause.

### Model development and interpretation

We employed LASSO regression for feature selection and systematically trained seven classical machine learning algorithms: LR, support vector machine (SVM), gradient boosting machine (GBM), neural network, XGBoost, k-nearest neighbors (KNN), and AdaBoost. To mitigate parameter sensitivity, hyperparameter optimization was performed *via* 10-fold cross-validation with grid search during training. During validation, optimal parameters were first screened using the AUC metric, then applied to the independent validation cohort. Beyond AUC, we established a comprehensive evaluation framework incorporating accuracy, sensitivity, specificity, precision, F1 score, Brier score, and calibration curves to assess model discrimination and calibration. SHapley Additive exPlanations (SHAP) values quantified feature importance by assigning each predictor an impact significance metric for individual predictions. Partial dependence plots (PDPs) visualized marginal feature effects on outcomes, enabling intuitive interpretation of predictor-outcome relationships and enhancing understanding of prediction mechanisms.

### Statistical analysis

All statistical analyses and visualizations were performed using R (version 4.4.2; R Foundation for Statistical Computing). Continuous variable normality was assessed using Shapiro–Wilk tests with Q–Q plot verification. Normally distributed variables are expressed as mean ± SD, while non-normally distributed variables are presented as median (interquartile range [IQR]). Categorical variables are reported as frequency counts and percentages (*n* [%]).

## Results

### Baseline characteristics

This study enrolled 387 patients, comprising 226 males (58.40%) and 161 females (41.60%), with a mean age of 65.44 ± 10.83 years. The cohort was stratified into non-difficult surgery (*n* = 300, 77.52%) and difficult surgery groups (*n* = 87, 22.48%). Statistically significant differences (*p* < 0.05) were observed between groups for BMI, tumor anal verge distance, ALB, NLR, SMI, SFA, VFA, prior abdominal surgery, and neoadjuvant therapy. No significant intergroup differences (*p* > 0.05) were detected in other parameters. Detailed baseline characteristics are presented in Table 2. Supplementary Table 1 demonstrates comparable distributions between training and validation sets with no significant differences observed (*p* > 0.05).

### Variable selection

**Figure 2.**
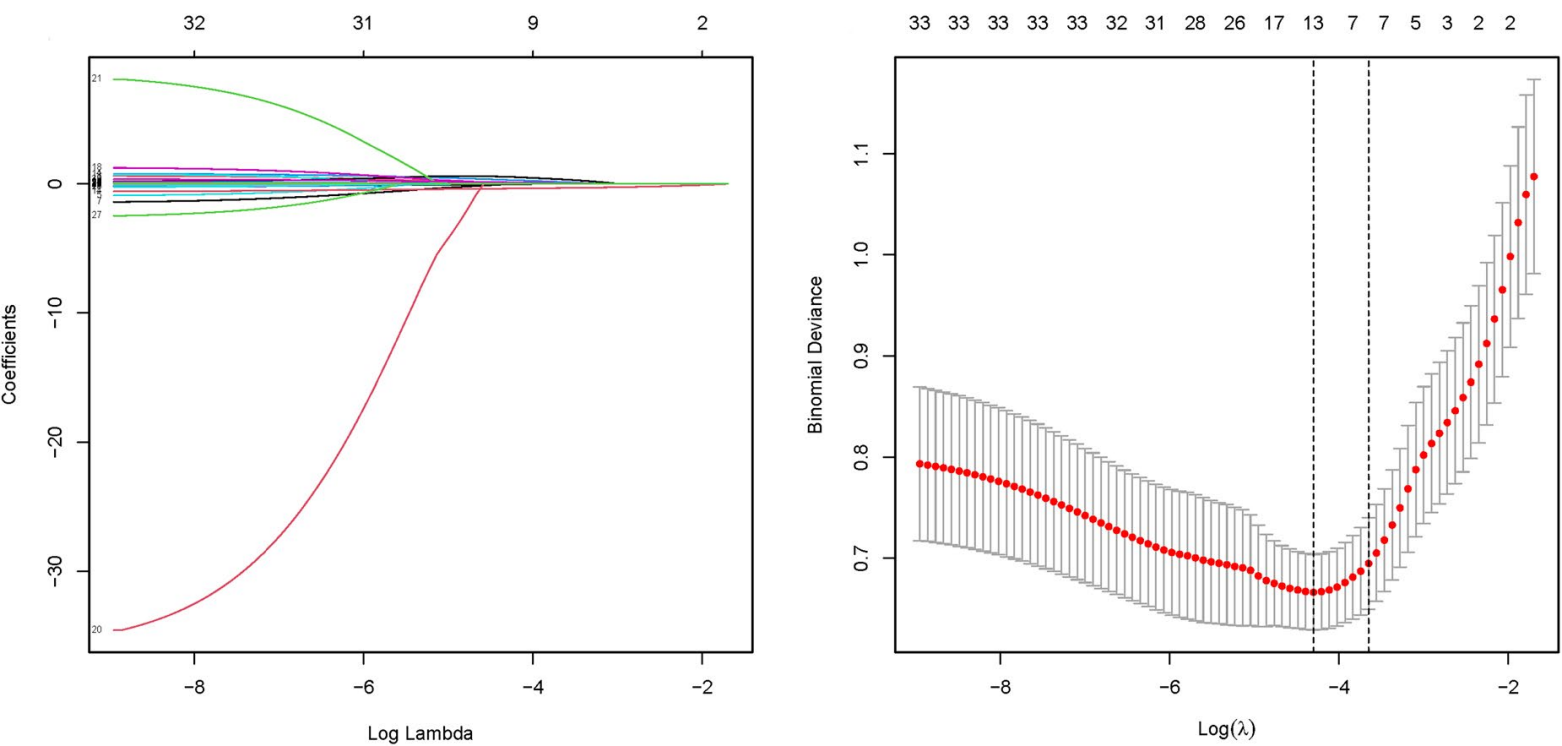
LASSO coefficient profiles for the 33 variables (A). Ten-fold cross-validation for selecting the optimal penalty coefficient (λ) in the LASSO model based on the minimum mean squared error criterion(B). Vertical dashed lines indicate lambda.min (left) and lambda.1se (right).

**Figure 3.**
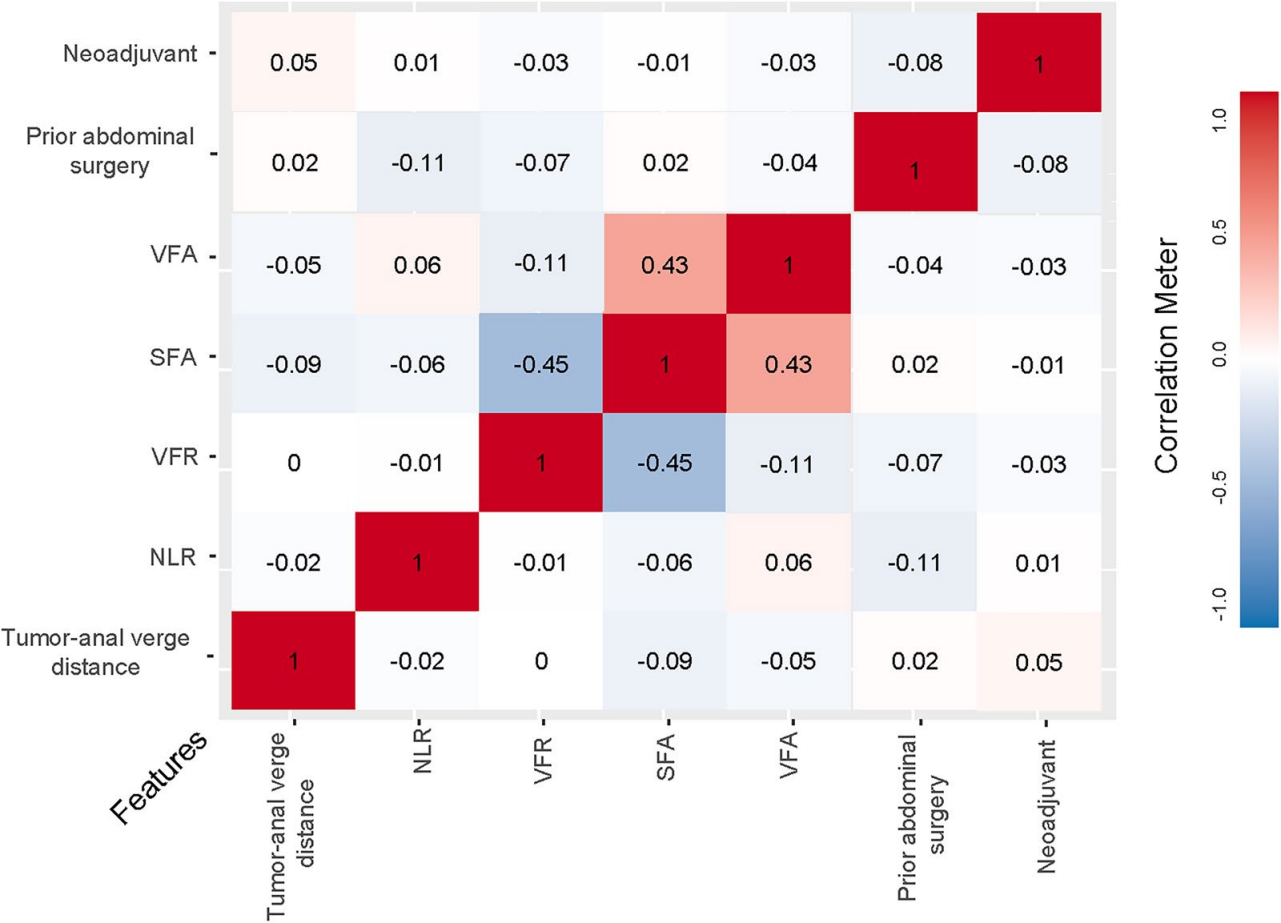
Feature correlation heatmap. Red indicates positive correlations, while blue represents negative correlations. Color intensity denotes the strength of correlation.

The LASSO coefficient path plot (Figure 2) displays two key lambda (λ) values: lambda.min and lambda.1se. While lambda.min yields a feature set, it may be large, resulting in a more complex model with reduced clinical utility. To enhance clinical applicability and interpretability, this study therefore selected the more parsimonious feature set corresponding to lambda.1se (λ = 0.03328) for model construction. The final seven selected features were: VFA, VFR, tumor anal verge distance, SFA, neoadjuvant therapy, NLR, and prior abdominal surgery. Their corresponding LASSO regression coefficients are presented in Table 3. Furthermore, all pairwise correlation coefficients between these features were below 0.5 (Figure 3), indicating the absence of significant multicollinearity.

**Table 3.** Performance of each model in the training set and validation set.

Model	Training set					
	Accuracy	Sensitivity	Specificity	Precision	Brier score	F1 score
Logistic	0.782	0.885	0.752	0.509	0.105	0.647
SVM	0.834	0.82	0.838	0.595	0.111	0.69
GBM	0.893	0.951	0.876	0.69	0.068	0.8
NeuralNetwork	0.845	0.869	0.838	0.609	0.091	0.716
Xgboost	0.871	0.918	0.857	0.651	0.079	0.762
KNN	0.911	0.902	0.914	0.753	0.081	0.821
Adaboost	0.9	0.934	0.89	0.713	0.161	0.809
	Validation set					
Logistic	0.853	0.808	0.867	0.636	0.102	0.712
SVM	0.75	0.885	0.711	0.469	0.109	0.613
GBM	0.724	0.923	0.667	0.444	0.132	0.6
NeuralNetwork	0.741	0.808	0.722	0.457	0.125	0.583
Xgboost	0.759	0.885	0.722	0.479	0.112	0.622
KNN	0.776	0.769	0.778	0.5	0.132	0.606
Adaboost	0.845	0.731	0.878	0.633	0.173	0.679

### Model development and validation

**Figure 4.**
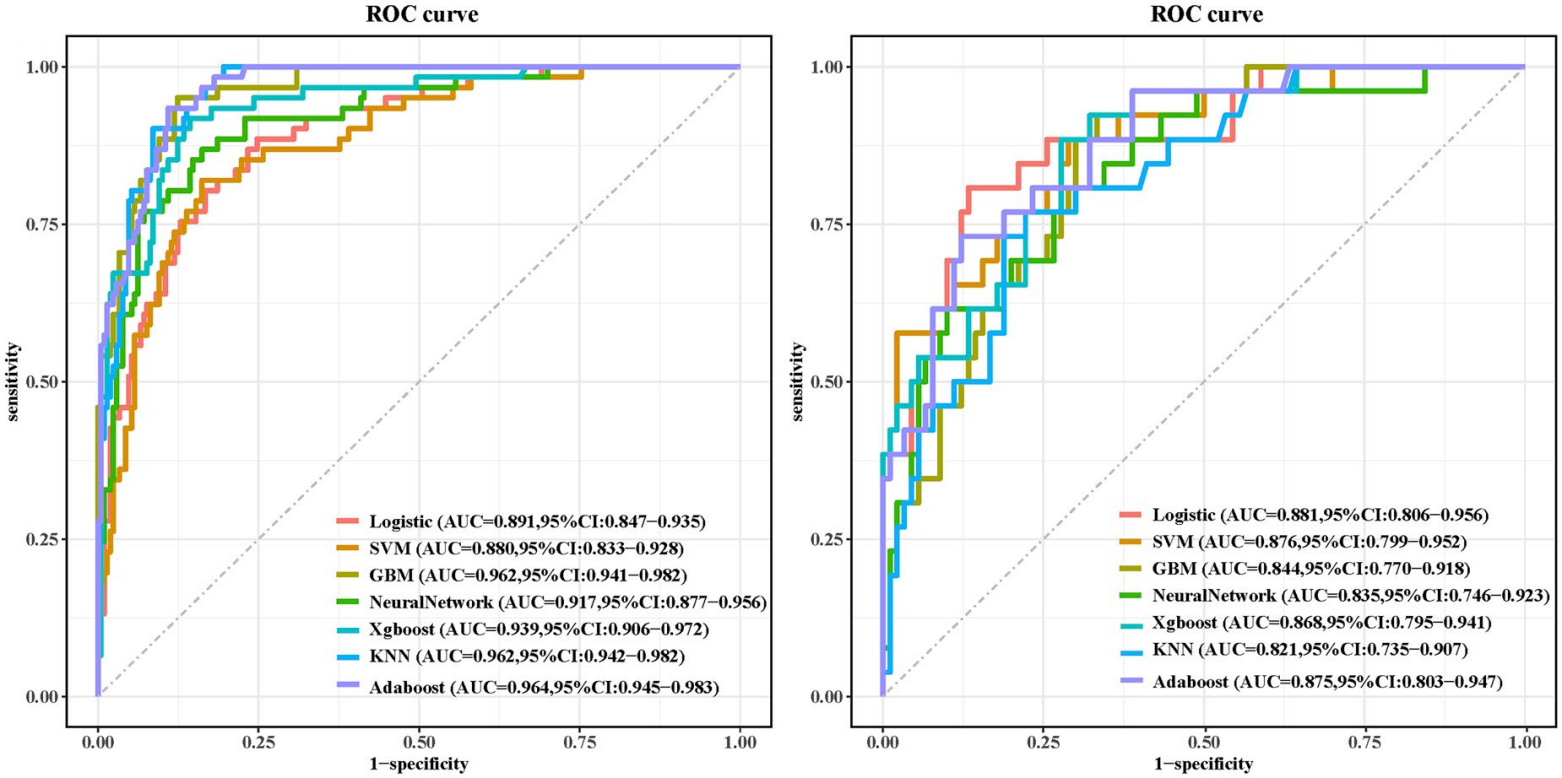
Performance of seven models measured by AUC in the training set (A) and validation set (B).

**Figure 5.**
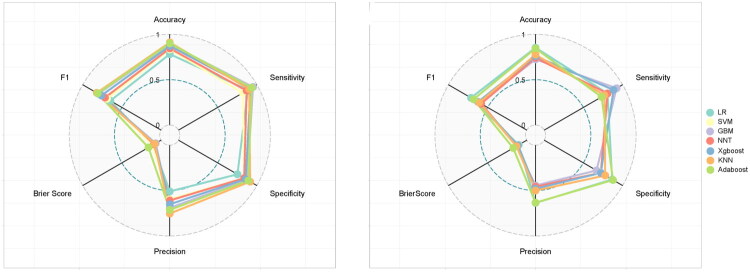
Radar chart visualizing performance metrics comparison across models. Training set radar chart (A); validation set radar chart (B).

**Figure 6.**
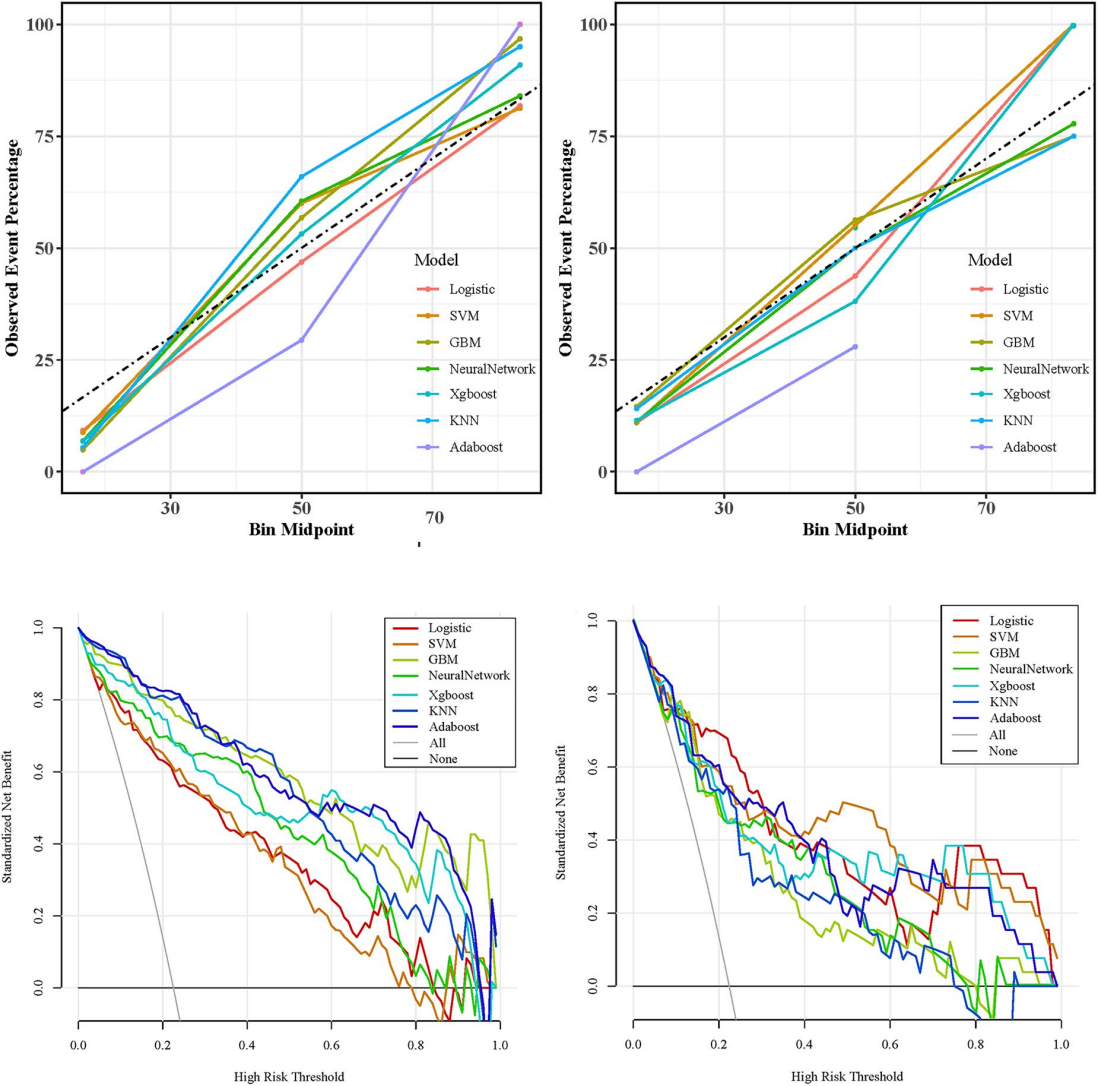
Calibration and decision curve analysis performance across models in training and validation sets. Training set calibration curve (A); validation set calibration curve (B); training set decision curve analysis (C); validation set decision curve analysis (D).

This study constructed a prediction model using the seven features selected by LASSO regression. To determine the optimal modeling strategy, we systematically compared multiple algorithms. During model development, we employed a combined strategy of 10-fold cross-validation and grid search for hyperparameter tuning: First, the optimal hyperparameter combination for each algorithm was identified within the training set by exhaustively searching the hyperparameter space, selecting the combination that maximized the AUC. Subsequently, the generalization performance of each model was assessed on an independent validation set. Finally, comprehensive indicator analysis was used to select the optimal algorithm. Among the seven models evaluated on the validation set, logistic regression (LR) achieved the highest AUC (0.881) (Figure 4), accuracy (0.853), precision (0.636), and F1 score (0.712), indicating strong overall performance and good calibration. GBM demonstrated the highest sensitivity (0.923), while AdaBoost exhibited the highest specificity (0.878) (Table 3). Furthermore, LR yielded the lowest Brier score (0.102). A radar chart comparing the evaluation metrics across all models is presented in Figure 5. Considering these multiple performance metrics, the LR model demonstrated superior predictive performance, maintaining high levels of both discrimination and calibration (Figure 6A,B). Regarding clinical applicability, decision curve analysis showed that all models provided robust net benefit across a wide range of clinically relevant threshold probabilities (Figure 6C,D). Given its overall advantages, the LR model was selected as the optimal algorithm. This model was subsequently utilized for interpretability analyses and the development of an interactive, web-based nomogram tool.

### Model interpretation

**Figure 7.**
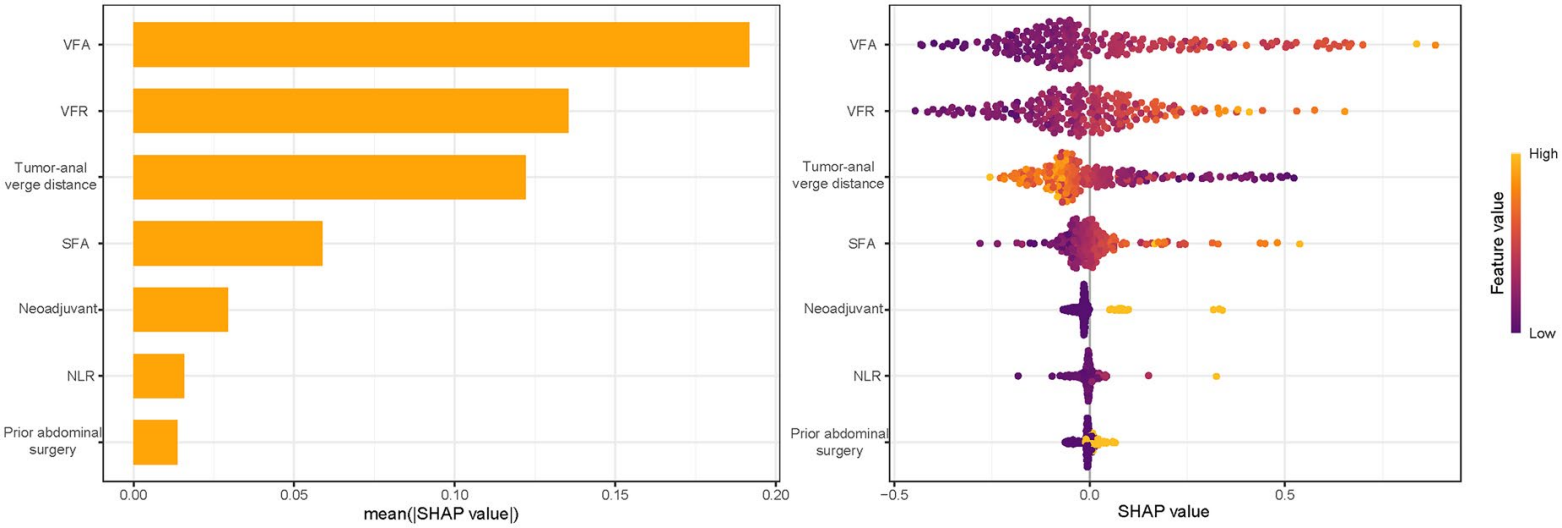
SHAP interpretability analysis of the LR Model. Feature importance ranking bar plot – bar length represents mean absolute SHAP values, indicating the global impact hierarchy of features on model predictions(A). Feature beeswarm plot – warm colors (yellow) denote high feature values, cool colors (purple) indicate low values, with horizontal dispersion revealing directional contributions of SHAP values to prediction outcomes (B).

**Figure 8.**
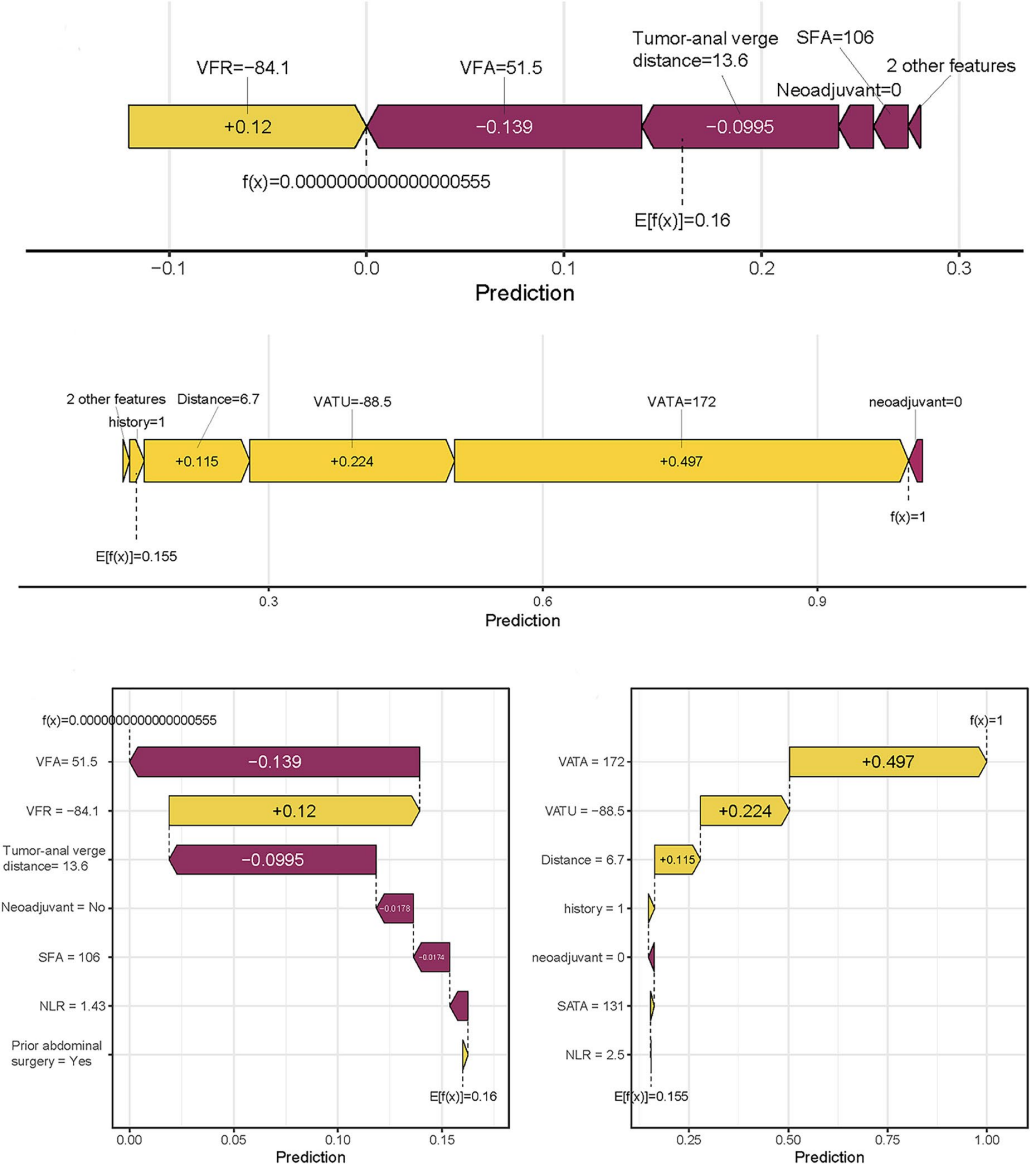
Force-directed graphs and waterfall plots from two patients. A and B display force-directed graphs based on clinical features of two individual patients; C and D present the corresponding waterfall plots for each patient, respectively.

**Figure 9.**
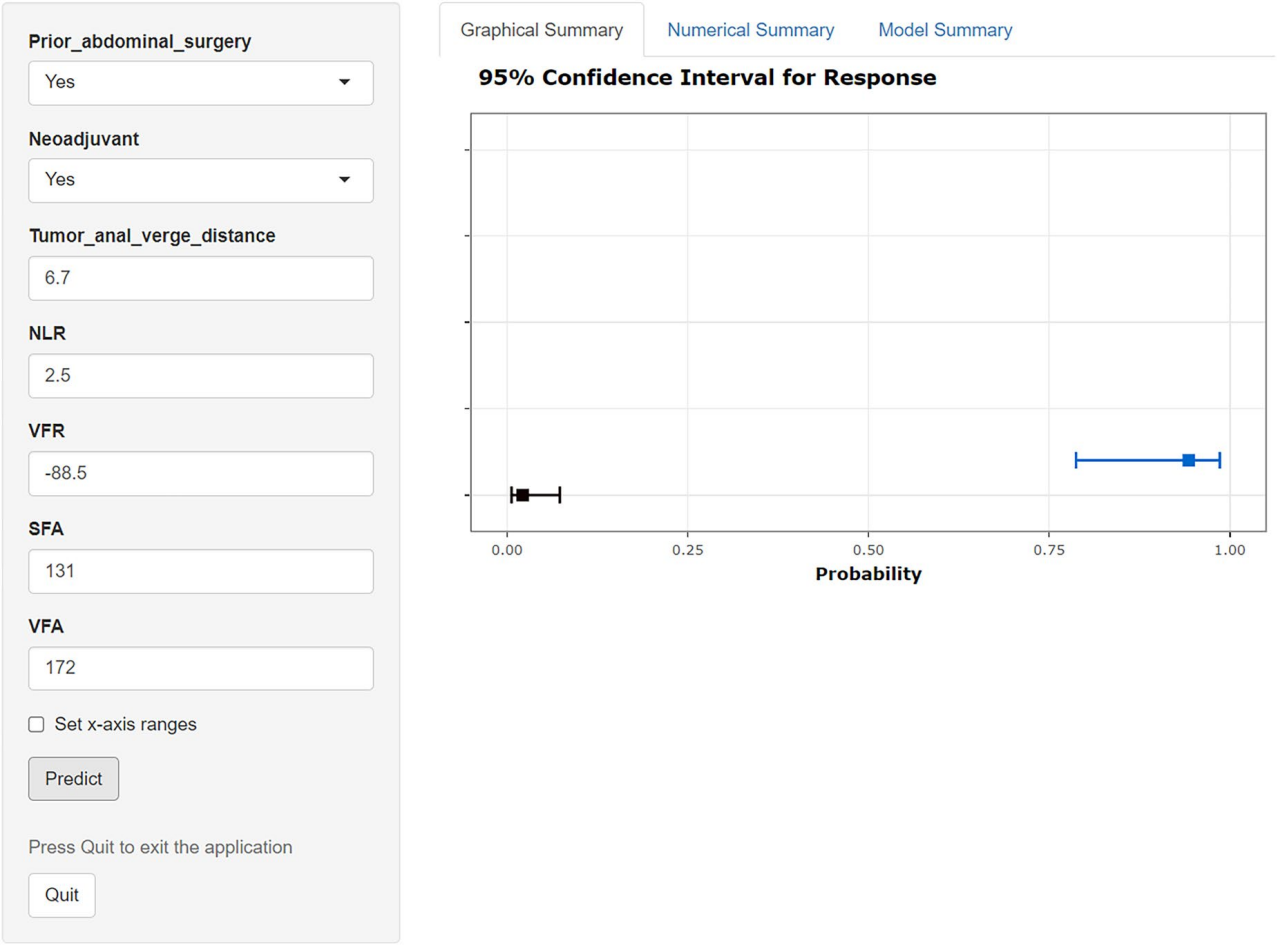
Interactive Shiny web-based prediction tool.

This study conducted comprehensive interpretability analysis of the LR model through feature importance assessment, SHAP value interpretation, force-directed graphs, waterfall plots, and PDPs to elucidate feature contributions to predictions. VFA demonstrated the highest SHAP value, establishing it as the most influential predictor of surgical difficulty, followed sequentially by VFR, tumor-to-anal-verge distance, SFA, neoadjuvant therapy, NLR, and abdominal surgery history (Figure 7). SHAP-based dependence plots revealed increasing SHAP values with higher VFA, indicating positive correlation with surgical difficulty, while elevated SFA and VFR similarly correlated with greater operative risk; conversely, SHAP values decreased with greater tumor-to-anal-verge distance, demonstrating an inverse relationship where proximal tumor location predicted higher surgical difficulty risk (Figure 8). For clinical implementation, we developed an interactive web-based prediction system (https://lixiangyong.shinyapps.io/dynnomapp/) enabling clinicians to input seven clinical features to estimate surgical difficulty probability during LaTME. As illustrated in two representative cases, the model generated surgical difficulty probabilities of 2.15% and 94.3% respectively (Figure 9).

### Survival analysis

**Figure 10.**
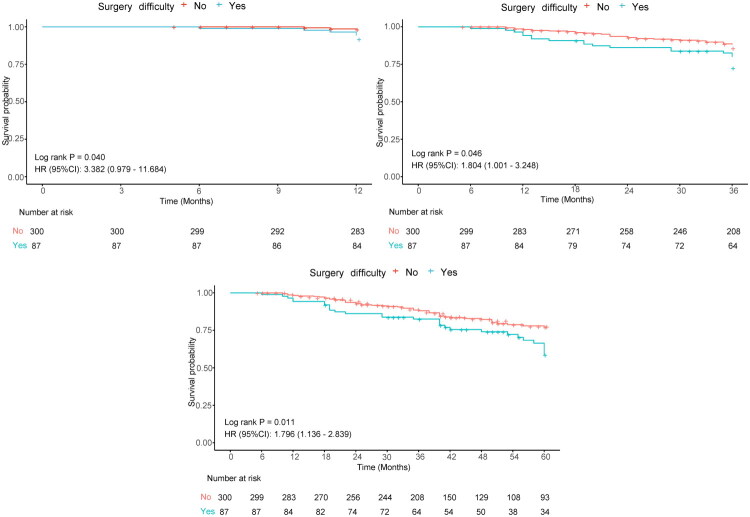
Comparison of Kaplan–Meier survival curves between low and high surgical difficulty patients at 1-year (A), 3-year (B), and 5-year (C) intervals. Blue curves denote the low surgical difficulty group; red curves represent the high surgical difficulty group.

Survival analysis was performed for the 387 enrolled patients (79 deaths). The median survival time for the entire cohort was 42 months. Kaplan–Meier curve comparison demonstrated consistently higher survival probabilities in the low surgical difficulty group compared to the high difficulty group at all time points (*p* < 0.05), with significantly better OS rates at 1, 3, and 5 years. These findings underscore the prognostic importance of surgical difficulty (Figure 10).

## Discussion

This study utilized a single-center cohort of 387 rectal cancer (RC) patients undergoing LaTME. Seven significant features were identified using LASSO regression: VFA, VFR, tumor anal verge distance, SFA, neoadjuvant therapy, NLR, and prior abdominal surgery. Optimal hyperparameters were obtained *via* 10-fold cross-validation with grid search on the training set and subsequently applied to the validation set. Seven distinct ML algorithms were constructed and validated. Their predictive performance was comprehensively compared across multiple metrics, including the AUC, sensitivity, specificity, F1 score, and Brier score. Ultimately, LR was identified as the optimal model. Feature importance ranking derived from the LR model highlighted VFA as the most influential feature affecting surgical difficulty. To facilitate clinical utility, a Shiny web application based on the LR prediction model was developed.

While LaTME has gained widespread adoption as the standard surgical procedure for RC, the association between its technical difficulty and patient body composition has not been systematically investigated. Previous studies consistently identified BMI as an indicator of obesity relevant to the development and prognosis of CRC [24,25]. However, conflicting reports exist regarding the relationship between obesity and mortality in cancer and several other chronic diseases [26–28]. The ‘obesity paradox’ refers to the phenomenon where overweight and mildly obese patients may exhibit a survival advantage. A potential explanation for this paradox is that BMI fails to accurately reflect fat distribution; consequently, BMI may be suboptimal for predicting surgical difficulty and outcomes. Excess abdominal visceral fat is likely the true factor impacting surgical difficulty and results. While numerous studies have confirmed the association between pelvic factors and TME surgical difficulty, few have focused on visceral fat. In fact, visceral fat is more intimately related to surgical difficulty. This is primarily because visceral obesity reduces the relative intra-abdominal space, leading to poor exposure (e.g. ill-defined tissue planes) and anatomical challenges due to thickened adipose tissue. Secondly, visceral fat adversely affects critical intraoperative maneuvers: dissecting between the visceral and parietal fascia, skeletonizing the rectal vessels, and managing smoke generated by ultrasonic scalpels. Furthermore, the larger mesenteric volume in obese patients predisposes them to mesenteric tears during retraction, potentially causing significant hemorrhage. This mesenteric tear-induced bleeding can be substantial enough to obscure the surgical field. Our findings demonstrate a proportional relationship between VFA and surgical difficulty, which aligns broadly with prior conclusions.

This study is the first to systematically reveal a significant association between elevated VFR and increased surgical difficulty in LaTME. The underlying pathophysiological mechanisms of this phenomenon may involve the dual effects of adipose microenvironment remodeling and systemic inflammatory responses. Previous studies have confirmed that adipose tissue radiodensity is not a constant parameter; its dynamic changes are closely linked to alterations in adipocyte morphology and the microenvironment. For instance, adipocyte size increases with weight gain, and higher adipocyte weight correlates with lower radiodensity. Consequently, lower radiodensity can be interpreted as a measure of adipocyte hypertrophy and higher lipid content [29]. While direct evidence linking increased VFR to surgical difficulty is currently lacking, it is closely associated with patient prognosis. A retrospective study proposed inflammation as a potential mechanistic link between adipose radiodensity and OS in cancer patients, suggesting that inflammation may also play a significant role in the relationship between VFR and surgical difficulty [30]. Secondly, increased radiodensity often indicates tissue edema, as the accumulation of protein-rich fluid (ascites) within the abdominal cavity elevates adipose tissue radiodensity. This accumulation may result from disease progression, potentially explaining why higher adipose radiodensity is often associated with increased surgical difficulty.

This study found that patients with a previous abdominal surgery experienced significantly increased LaTME surgical difficulty, primarily due to the persistent impact of adhesive pathology on anatomical planes. Clinical data indicate that approximately 90% of patients undergoing abdominal surgery develop varying degrees of intra-abdominal adhesions, with incidence positively correlating with the number of prior surgeries [31,32]. However, the impact of prior abdominal surgery on LaTME remains contentious. Traditional views hold that a surgical history significantly prolongs operative time and increases intraoperative blood loss. Nevertheless, large-scale clinical studies show no statistically significant difference in operative time (182 ± 45 vs. 175 ± 40 min, *p* > 0.05) or blood loss (150 ml vs. 140 ml, *p* > 0.05) between patients with prior abdominal surgery and controls [33]. Furthermore, only 28.6% of studies (5/17) reported significantly prolonged operative time [34].

This study identifies neoadjuvant therapy as another significant factor influencing surgical difficulty, with patients receiving neoadjuvant therapy experiencing higher difficulty than those who did not. Neoadjuvant therapy is a current standard treatment enabling surgery for locally advanced RC patients, has been proven to reduce local recurrence and improve survival rates [35]. It significantly reduces tumor size and improves exposure in the surgical field, facilitating the achievement of safe resection margins [36]. However, successful neoadjuvant therapy-induced tumor shrinkage or complete clinical response makes intraoperative localization of the primary tumor more challenging. Following tumor regression, accurate determination of the original distal tumor margin *via* intraoperative digital rectal examination may be impossible. Despite various preoperative localization techniques, regressed tumors can be mistaken for polyps, increasing localization difficulty and operative time [37]. Our study also identified operative time as a key indicator of surgical difficulty, consistent with this finding. Additionally, preoperative chemoradiotherapy can cause tissue edema, excessive surgical smoke, and effusion during laparoscopic RC resection, hindering tissue dissection and potentially increasing operative time and intraoperative blood loss.

Furthermore, this study incorporated several common biomarkers: NLR, IBI, NAR, and LCR. Tumor-associated inflammation is widely recognized as the immune system’s response to tumor cells. Uncontrolled inflammation is closely linked to tumor initiation, progression, invasion, and metastasis [38,39]. We selected biomarkers previously demonstrated to play significant roles in RC development and progression [40–42]. Sun et al. in a study of 294 locally advanced RC patients undergoing LaTME, found that the preoperative albumin-to-globulin ratio could predict surgical difficulty post-CRT, highlighting the importance of preoperative nutritional status in assessing difficulty [43]. In our study, following feature variable selection *via* LASSO regression, only NLR was identified as a predictor of surgical difficulty. A meta-analysis encompassing 959 RC patients from 7 studies evaluated the prognostic value of NLR, finding that elevated NLR was significantly associated with worse OS (HR = 13.408), disease-free survival (HR = 4.368), and recurrence-free survival (RFS) (HR = 3.636) [44]. These findings suggest that serum biomarkers, as key components of body composition, are cost-effective and readily accessible. However, few studies have explored the relationship between inflammatory serum biomarkers and surgical difficulty. This study demonstrates an association between higher NLR and increased surgical difficulty, a novel finding with mechanisms requiring further investigation.

In our study, a low tumor location emerged as a significant predictor of surgical difficulty in patients undergoing LaTME. The distance between the inferior tumor border and the anal verge is a key determinant for performing TME and accessing the deep pelvis. RC surgery is conducted through the narrow, funnel-shaped bony pelvic inlet, which inherently complicates deep pelvic access and visualization. Even with a relatively straightforward open approach, maintaining a clear operative field, identifying precise anatomical structures, and performing accurate rectal resection while preserving genitourinary function present substantial challenges. Furthermore, a lower tumor position increases the technical difficulty of rectal transection and anastomosis.

Tumors located closer to the anal verge require more extensive dissection and exposure, significantly elevating the risk of hemorrhage. This heightened risk is directly attributable to an increased probability of injuring the venous plexus during retrograde mesorectal dissection.

This study further demonstrated that patients in the low surgical difficulty group had significantly better prognoses than those in the high difficulty group. Although no similar studies have reported identical conclusions to date, this finding aligns with the results of the multicenter study by Yu et al. on predicting surgical difficulty in laparoscopic right hemicolectomy [45]. That study, utilizing both internal and external validation sets, confirmed significantly superior 3-year OS in the low difficulty group compared to the high difficulty group. Further analysis suggests this survival disparity may be closely linked to tumor anatomical location and the associated risk of postoperative anastomotic leakage. Existing literature indicates that tumor location not only correlates with recurrence risk but is also a critical prognostic factor for long-term survival [46,47]. Anatomical studies confirm that primary anastomosis performed at low levels carries a significantly increased risk of anastomotic leakage, which multiple studies have established as an independent risk factor for reduced OS [48]. This suggests that the poorer prognosis observed in the high surgical difficulty group may pathophysiologically stem from the technical challenges posed by low rectal tumors and the consequent increased risk of postoperative complications.

Previous research has primarily focused on investigating the impact of individual or combined factors such as VFA or tumor anal verge distance on surgical difficulty. Although some studies have attempted to construct predictive models using visual tools like nomograms, significant limitations remain regarding clinical convenience and practical utility. This study innovatively integrates an LR-based Shiny interactive web application with the SHAP interpretability framework, creating a predictive system with dynamic visualization capabilities. This approach maintains predictive performance while substantially enhancing the model’s clinical applicability. These two technologies each demonstrated unique advantages and limitations during the modeling process.

Despite achieving excellent predictive performance and visualization through the construction of an ML algorithm system for retrospective analysis of LaTME surgical difficulty, we must acknowledge several limitations inherent in this study: A noteworthy limitation is the absence of external validation. Its single-center retrospective design (introducing potential selection bias) and reliance on CT data (measured by surgeons, lacking pelvic parameter analysis) may limit model generalizability. Critical prognostic factors such as lifestyle habits and family history were missing from the electronic health record data. Feature selection might have omitted relevant variables, and model construction (employing only 7 ML algorithms) did not exhaustively explore optimal algorithms/architectures. Although the model demonstrated favorable predictive performance, it is noteworthy that the imbalance in the enrolled patient cohort may affect its predictive capabilities.

## Conclusions

In summary, this study successfully established an ML algorithm-based predictive model for surgical difficulty in LaTME. The LR algorithm demonstrated optimal overall performance. VFA, VFR, distance from the tumor to the anal verge, SFA, NLR and history of prior abdominal surgery were identified as significant features for predicting LaTME difficulty. Furthermore, a web-based deployment of the model has been completed.

## Supplementary Material

Supplementary.docx

## Data Availability

The data sets analyzed during the current study are not publicly available for patient privacy purposes but are available from the corresponding author upon reasonable request (Wu).

## Supplementary information

Supplementary Figure S1. Consecutive CT scans at the L3 level (A,B) and corresponding segmentation diagrams (C,D).

Supplementary Table 1. Comparative analysis of baseline characteristics between training and validation cohorts.

## Abbreviations

LaTME: Laparoscopic total mesorectal excision
LR: Logistic regression
SVM: Support vector machine
GBM: Gradient boosting machine
Xgboost: Extreme Gradient Boosting
KNN: K-nearest neighbors
Adaboost: Adaptive boosting
SHAP: SHapley Additive exPlanations
PDP: Partial dependence plot
VFA: Visceral fat area
VFR: Visceral fat radiodensity
SFA: Subcutaneous fat area
NLR: Neutrophil-to-lymphocyte ratio
CRC: Colorectal cancer
RC: Rectal cancer
TME: Total mesorectal excision
CRM: Circumferential resection margin
BMI: Body mass index
CT: Computed tomography
IBI: Inflammatory burden index
NAR: Neutrophil-to-albumin ratio
LCR: Lymphocyte-to-C-reactive protein ratio
ML: Machine learning
AI: Artificial intelligence
ASA: American Society of Anesthesiologists
Alb: Albumin
Tg: Triglycerides
TC: Total cholesterol
CRP: C-reactive protein
CEA: Carcinoembryonic antigen
SM: Skeletal muscle
OS: Overall survival
