# Supplementary material for: Machine learning combined with body composition predicts surgical difficulty in mid-low rectal cancer surgery
Source: Ann Med. 2025 Nov 17;57(1):2582235. doi: 10.1080/07853890.2025.2582235 (PMC12624905; doi:10.1080/07853890.2025.2582235)
Supplement: Supplementary.docx [file IANN_A_2582235_SM6055.docx]

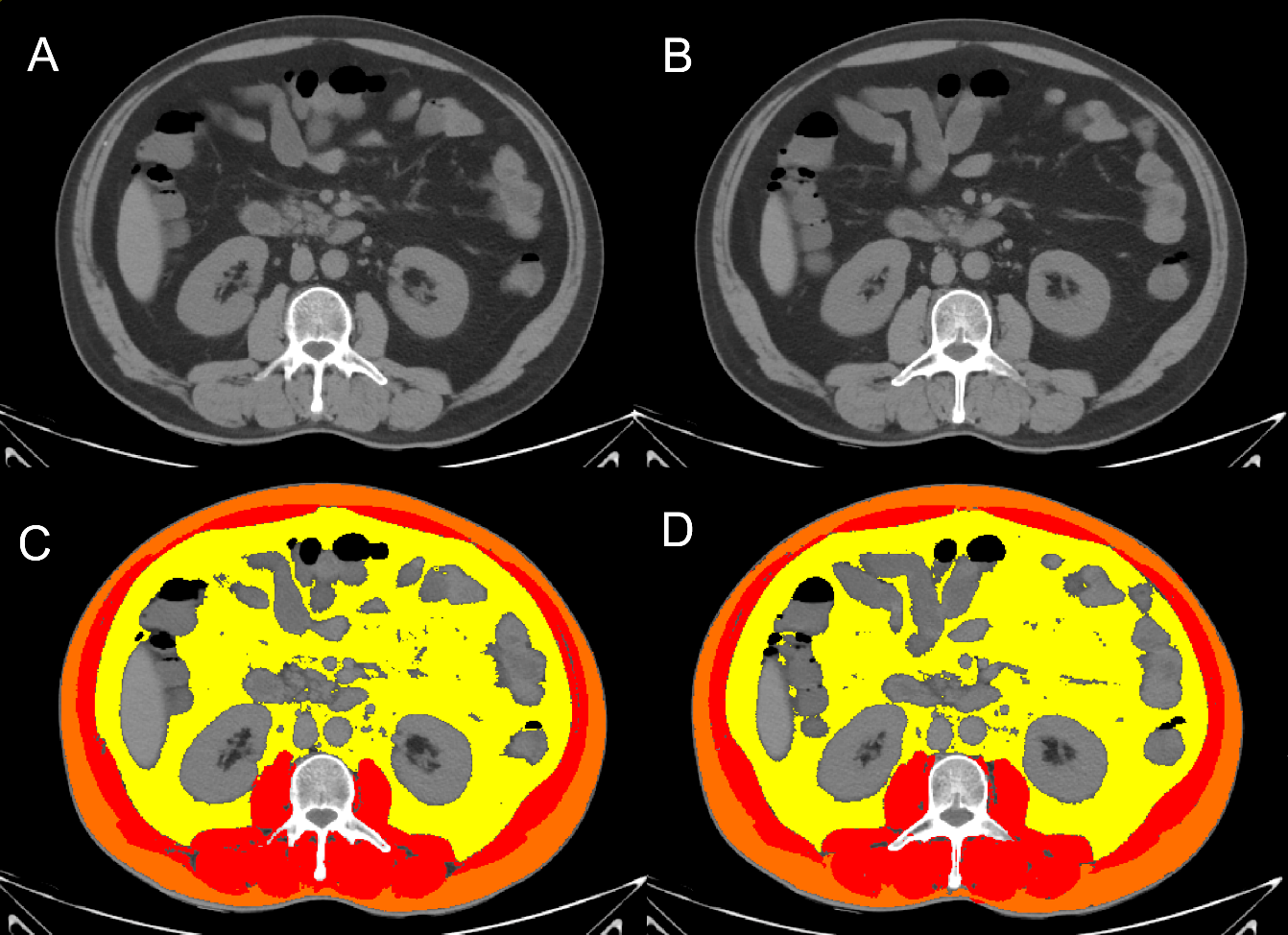


**Supplementary Figure S1.** Consecutive CT scans at the L3 level (A-B) and corresponding segmentation diagrams (C-D). Orange represents subcutaneous adipose tissue, yellow indicates visceral adipose tissue, and red denotes skeletal muscle tissue. This demonstrates body composition measurements in a 52-year-old male patient (BMI=31.2 kg/m²). Quantification revealed the following parameters across both slices:

Visceral Fat Area (VFA): 296 cm² and 299.3 cm²; Visceral Fat Radiodensity (VFR): -104 HU and -102.7 HU; Subcutaneous Fat Area (SFA): 42.1 cm² and 41.5 cm²; Subcutaneous Fat Radiodensity (SFR): -100.70 HU and -100.4 HU; Skeletal Muscle Area (SMA): 147.1 cm² and 142.7 cm²; Skeletal Muscle Radiodensity (SMR): 37.9 HU and 37.35 HU. The patient's final computed metrics were: VFA: 297.65 cm²; VFR: -103.35 HU; SFA: 41.8 cm²; SFR: -100.55 HU; SMA: 144.9 cm²; SMR: 37.625 HU.

**Supplementary Table 1**. Comparative Analysis of Baseline Characteristics Between Training and Validation Cohorts

| **Characteristics** | **Total（n=387）** | **Training set（n=271）** | **validation set（n=116）** | ***P*** |
| --- | --- | --- | --- | --- |
| **Baseline Characteristics** | | | | |
| Age (years), mean [SD] | 65.44 ± 10.83 | 65.21 ± 10.28 | 65.98 ± 12.05 | 0.523 |
| BMI (kg/m^2^), mean [SD] | 23.73 ± 3.71 | 23.73 ± 3.50 | 23.72 ± 4.19 | 0.982 |
| Sex, n(%) |  |  |  | 0.867 |
| Female | 161（41.60） | 112（41.33） | 49（42.24） |  |
| Male | 226（58.40） | 159（58.67） | 67（57.76） |  |
| Hypertension, n(%) |  |  |  | 0.513 |
| No | 200（51.68） | 143（52.77） | 57（49.14） |  |
| Yes | 187（48.32） | 128（47.23） | 59（50.86） |  |
| Diabetes mellitus, n(%) |  |  |  | 0.601 |
| No | 326（84.24） | 230（84.87） | 96（82.76） |  |
| Yes | 61（15.76） | 41（15.13） | 20（17.24） |  |
| Prior of abdominal surgery, n(%) |  |  |  | 0.974 |
| No | 294（75.97） | 206（76.01） | 88（75.86） |  |
| Yes | 93（24.03） | 65（23.99） | 28（24.14） |  |
| Neoadjuvant therapy, n(%) |  |  |  | 0.086 |
| No | 349（90.18） | 249（91.88） | 100（86.21） |  |
| Yes | 38（9.82） | 22（8.12） | 16（13.79） |  |
| ASA, n(%) |  |  |  | 0.009 |
| 1 | 138（35.66） | 100（36.90） | 38(32.76) |  |
| 2 | 154（39.79） | 117（43.17） | 37(31.90) |  |
| 3 | 78（20.16） | 43（15.87） | 35(30.17) |  |
| 4 | 17（4.39） | 11（4.06） | 6(5.17) |  |
| **Laboratory Parameters** | | | | |
| Hb(g/L), mean [SD] | 128.90 ± 19.59 | 129.18 ± 19.67 | 128.24 ± 19.45 | 0.666 |
| Alb(g/L), mean [SD] | 41.41 ± 4.92 | 41.33 ± 4.89 | 41.59 ± 4.99 | 0.635 |
| NLR, mean [SD] | 3.09 ± 3.89 | 3.08 ± 3.58 | 3.10 ± 4.57 | 0.974 |
| IBI, median [IQR] | 12.57（9.21,18.84） | 12.47（9.07,18.29） | 12.96（9.40,19.96） | 0.244 |
| NAR, median [IQR] | 0.08（0.06,0.11） | 0.08（0.06,0.11） | 0.08（0.06,0.10） | 0.749 |
| LCR, median [IQR] | 0.26（0.19,0.36） | 0.26（0.20,0.36） | 0.27（0.17,0.35） | 0.505 |
| Tc(mmol/L), median [IQR] | 4.59（4.12,5.22） | 4.59（4.15,5.22） | 4.55（4.01,5.22） | 0.55 |
| Tg(mmol/L), median [IQR] | 1.18（0.91,1.54） | 1.17（0.90,1.54） | 1.21（0.97,1.53） | 0.486 |
| CRP, median [IQR] | 5.40（5.10,5.70） | 5.40（5.10,5.70） | 5.50（5.10,5.80） | 0.333 |
| CEA(ng/ml), median [IQR] | 3.34（2.21,6.15） | 3.68（2.34,6.27） | 3.04（1.86,5.92） | 0.075 |
| **Quantitatively Measured CT Parameters** | | | | |
| SMR(HU), mean [SD] | 32.78 ± 7.03 | 32.80 ± 7.18 | 32.74 ± 6.69 | 0.938 |
| SFR(HU), mean [SD] | -97.40 ± 9.73 | -97.71 ± 9.13 | -96.69 ± 11.02 | 0.348 |
| VFR(HU), mean [SD] | -92.99 ± 8.07 | -93.15 ± 7.49 | -92.59 ± 9.31 | 0.566 |
| SMA(cm^2^), mean [SD] | 118.81 ± 26.41 | 119.19 ± 26.45 | 117.93 ± 26.41 | 0.668 |
| SFA(cm^2^), mean [SD] | 124.31 ± 57.91 | 124.80 ± 54.71 | 123.16 ± 65.00 | 0.799 |
| VFA(cm^2^), mean [SD] | 108.82 ± 58.87 | 109.72 ± 56.86 | 106.71 ± 63.54 | 0.645 |
| Tumor Distance from the Anal Verge  (cm), mean [SD] | 8.64 ± 2.91 | 8.64 ± 2.93 | 8.62 ± 2.87 | 0.953 |
| **Pathological Characteristics** | | | | |
| Differentiation grade,(n(%)) |  |  |  | 0.128 |
| Well-differentiated | 15（3.88） | 12（4.43） | 3（2.59） |  |
| Moderately differentiated | 313（80.88） | 212（78.23） | 101（87.07） |  |
| Poorly differentiated | 59（15.25） | 47（17.34） | 12（10.34） |  |
| Perineural invasion,(n(%)) |  |  |  | 0.072 |
| No | 316（81.65） | 215（79.34） | 101（87.07） |  |
| Yes | 71（18.35） | 56（20.66） | 15（12.93） |  |
| Lymphovascular invasion, (n(%)) |  |  |  | 0.668 |
| No | 325（83.98） | 229（84.50） | 96（82.76） |  |
| Yes | 62（16.02） | 42（15.50） | 20（17.24） |  |
| T stage, (n(%)) |  |  |  | 0.557 |
| 1 | 150（38.76） | 102（37.64） | 48（41.38） |  |
| 2 | 90（23.26） | 62（22.88） | 28（24.14） |  |
| 3 | 119（30.75） | 89（32.84） | 30（25.86） |  |
| 4 | 28（7.24） | 18（6.64） | 10（8.62） |  |
| N stage, (n(%)) |  |  |  | 0.194 |
| 0 | 240（62.02） | 163（60.15） | 77（66.38） |  |
| 1 | 86（22.22） | 67（24.72） | 19（16.38） |  |
| 2 | 61（15.76） | 41（15.13） | 20（17.24） |  |
| TNMstage, (n(%)) |  |  |  | 0.469 |
| I | 164（42.38） | 110（40.59） | 54（46.55） |  |
| II | 76（19.64） | 53（19.56） | 23（19.83） |  |
| III | 147（37.98） | 108（39.85） | 39（33.62） |  |
| LNR, median [IQR] | 0.00（0.00,0.13） | 0.00（0.00,0.13） | 0.00（0.00,0.11） | 0.358 |
| Tumor size(cm), mean [SD] | 37.51 ± 15.09 | 37.97 ± 14.65 | 36.46 ± 16.09 | 0.368 |

VFA, Visceral Fat Area VFR Visceral Fat Radiodensity; SFA, Subcutaneous Fat Area; NLR, Neutrophil-to-Lymphocyte Ratio;CT, Computed Tomography; IBI, Inflammatory Burden Index; NAR, Neutrophil-to-Albumin Ratio; LCR, Lymphocyte-to-C-reactive Protein Ratio; ASA, American Society of Anesthesiologists; Alb, Albumin; Tg, Triglycerides; TC, Total Cholesterol; CRP, C-Reactive Protein; CEA Carcinoembryonic Antigen; SM, Skeletal Muscle.
